# Is Cardiopulmonary Exercise Testing Predictive of Survival Outcomes in Patients Undergoing Surgery for Ovarian Cancer? †

**DOI:** 10.3390/cancers17091460

**Published:** 2025-04-26

**Authors:** Velangani Bhavya Swetha Rongali, Joanne Knight, Chloe Banfield, Porfyrios Korompelis, Stuart Rundle, Anke Smits

**Affiliations:** 1 Northern Gynecological Oncology Centre, Queen Elizabeth Hospital, Gateshead NE9 6SX, UK; porfyrios.korompelis@nhs.net (P.K.); stuart.rundle@nhs.net (S.R.); anke.smits1@nhs.net (A.S.); 2 Department of Anaesthetics, Queen Elizabeth Hospital, Gateshead NE9 6SX, UK; joanne.knight5@nhs.net; 3 School of Medicine, Newcastle University, Newcastle NE2 4HH, UK; chloe.banfield@swft.nhs.uk

**Keywords:** ovarian cancer, cardiopulmonary exercise testing, survival, prehabilitation, fitness

## Abstract

Preoperative cardiopulmonary exercise testing (CPET) provides an objective measure of a patient’s functional capacity under stress. However, the association between CPET and long-term outcomes for women with ovarian cancer have not been assessed. The aim was to determine whether cardiorespiratory fitness, as measured by CPET parameters—peak oxygen uptake (VO_2_ peak), ventilatory efficiency at anaerobic threshold (VE/VCO_2_ at AT), and anaerobic threshold (AT)—could predict overall survival (OS) and recurrence -free survival (RFS) in patients with all stages of ovarian cancer. Methods: This was a retrospective cohort study of patients who underwent CPET prior to surgery for suspected or confirmed ovarian cancer during 2019–2023 at the Northern Gynaecological Oncology Centre, United Kingdom. CPET outcomes were risk-stratified, with thresholds of AT ≥ 10 mL/min, VO_2_ peak ≥ 15 mL/kg/min, and VE/VCO_2_ at AT ≤ 34 indicating lower risk. Primary outcomes included OS and RFS. Results: A total of 303 patients were included, of whom 56 (18.5%) had a staging laparotomy, 130 (42.9%) underwent primary cytoreductive surgery, and 117 (38.6%) underwent interval cytoreductive surgery. Survival analysis showed that VO_2_ peak ≥ 15 was significantly associated with improved OS of the whole population (*p* = 0.032). VE/VCO_2_ at AT ≤ 34 was associated with improved survival in patients with advanced stage disease (*p* = 0.025) after ovarian cancer surgery. There was no association between CPET parameters and RFS. Conclusions: We found that peak VO_2_ ≥ 15 was associated with improvement of overall survival in patients with all stages of ovarian cancer. In addition, VE/VCO_2_ at AT ≤ 34 was associated with overall survival in patients with advanced-stage disease.

## Simple Summary

Treatment of ovarian cancer involves a combination of extensive surgery and chemotherapy. Due to the impact of ovarian cancer on a patient’s physical wellbeing and nutritional status, this population is usually characterised by poor physical fitness. Preoperative cardiopulmonary exercise testing (CPET) is used to assess patients’ ability to withstand the stress of an extensive surgery. In addition, CPET has also been recognised as a tool to predict survival outcomes after various cancer surgeries, but this has not yet been evaluated for patients with ovarian cancer. The aim of our study was to evaluate the value of CPET in predicting overall and recurrence-free survival in patients undergoing ovarian cancer surgery. We found that patients with a higher VO_2_ Peak ≥ 15 and a lower VE/VCO_2_ at AT ≤ 34 have longer overall survival. We did not find any relation between CPET and disease recurrence. We believe that improving cardiovascular fitness may play a role in improving survival in ovarian cancer patients.

## 1. Introduction

Ovarian cancer is the sixth most common cancer among women in the UK, with approximately 7500 new cases annually [1,2]. Women with ovarian cancer are usually characterised by increasing age, with a peak incidence between 75 and 79 years, poor performance status, and high symptom burden [1,2,3]. Most ovarian cancer patients are diagnosed at an advanced stage, and treatment usually comprises a combination of surgery and chemotherapy [1]. The surgical procedures are typically extensive, with the aim of removing all visible disease [1,4,5]. Despite, maximal surgical effort survival remains poor, with an overall mortality rate of 32% within the first year, with patient characteristics such as performance status and patient frailty having been recognised as important predictors of survival [2,5,6].

Patients with ovarian cancer are regarded as high risk for surgery due to the extensive disease burden and its detrimental effect on their nutritional status and physical fitness [2,7,8,9]. Recent data from the ovarian cancer audit feasibility pilot showed that 26.2% of women with ovarian cancer were not offered treatment due to poor fitness levels [1,3]. Fitness is commonly assessed using clinical frailty scores and other objective measures of preoperative physical fitness such as cardiopulmonary exercise testing (CPET), walk tests, and grip strength assessments [1,10].

Over the last few years, CPET has gained traction in the evaluation of a patient’s fitness prior to surgery. CPET evaluates the patient’s pulmonary and cardiac systems through ECG, lung function tests, blood pressure monitoring, continuous oxygen saturation assessment, and measurements of inspired and expired gases during exercise [11,12]. It provides an objective measure of a patient’s functional capacity under stress and is frequently used to determine their ability to withstand the rigours of extensive surgery. Recent studies have shown that some parameters of CPET may predict postoperative morbidity in ovarian cancer and other types of cancer surgery [8,13,14]. Studies of patients with non-gynaecological tumours undergoing radical surgical resection showed an association between CPET parameters and survival, but similar data for women being treated surgically for ovarian cancer are lacking [15]. As there is emerging evidence on the relationship between cardiorespiratory fitness and survival following cancer diagnosis, CPET may offer valuable prognostic insights in addition to assessing preoperative risks and aiding surgical planning in women with ovarian cancer [16]. In addition, pre-treatment identification of patients at risk of poorer prognosis due to decreased fitness may provide a window of opportunity for optimisation of these patients to improve long-term survival.

At the Northern Gynaecological Oncology Centre (NGOC), a high-volume ovarian cancer surgical centre and European Society of Gynaecological Oncology (ESGO)-accredited centre of excellence for ovarian cancer surgery, CPET assessment is standard care prior to staging and cytoreductive surgery for ovarian cancer for all patients. The primary aim of this study is to determine whether cardiorespiratory fitness, as measured by CPET parameters, can predict overall and progression-free survival in patients with all stages of ovarian cancer, who are undergoing surgery as part of their treatment.

## 2. Materials and Methods

### 2.1. Study Population

This was a retrospective cohort study of patients who underwent CPET as part of their preoperative anaesthetic assessment for suspected or confirmed ovarian cancer between January 2019 and January 2023 at the Northern Gynaecological Oncology Centre (NGOC), United Kingdom. CPET was offered to all patients, as a standard part of pre-operative anaesthetic assessment. Patients were excluded if they had a final benign or borderline histological diagnosis or if CPET was not performed following relative or absolute contra-indications [12]. All the patients in this study underwent an exploratory laparotomy and peritoneal evaluation. In those without a presurgical diagnosis of ovarian cancer, frozen-section analysis of the mass lesion was used for intraoperative diagnosis to guide surgical management [17]. When malignancy was confirmed through frozen-section analysis, a staging procedure was performed, which included total hysterectomy, bilateral salpingo-oophorectomy, infracolic omentectomy, and systematic pelvic lymphadenectomy with para-aortic lymphadenectomy. In cases with pre-operative histological or radiological diagnosis of peritoneal spread, maximal effort cytoreductive surgery was carried out in either the primary setting, prior to adjuvant chemotherapy, or as an interval procedure after neo-adjuvant chemotherapy according to ESGO guidance and frailty [18]. Ethical committee approval was exempted as this study is a part of an established continuous audit of practice and service evaluation at a referral centre for ovarian cancer.

### 2.2. Data Collection

Retrospective data collection was performed using medical records. Baseline characteristics included age at diagnosis, body mass index (BMI), the American Society of Anaesthesiologists (ASA) physical status, medical comorbidities according to the Charlson Comorbidity Index (CCI), performance status according to the Eastern Cooperative Oncology Group (ECOG), and smoking status [19]. Clinical characteristics included FIGO stage, grade, type of surgery, timing of surgery (primary or interval), and outcomes of cytoreductive surgery—complete (no macroscopic residual disease), optimal (residual disease ≤ 1 cm), or suboptimal cytoreduction (residual disease ≥ 1 cm) [8,20].

CPET was performed according to Perioperative Exercise Testing and Training Society (POETTS) guidelines and interpreted by consultant anaesthetists trained through POETTS [12]. The Ergoselect 200 (electromagnetically braked cycle ergometer) and CardiO2 System metabolic cart were used for exercise testing and to measure ventilation and gas exchange, respectively. Resting spirometry was routinely performed, except between March 2020 and July 2022, due to concerns regarding aerosol-generating procedures during the SARS-CoV-2 pandemic [8]. The CPET parameters measured included anaerobic threshold (AT, mL/kg/min), peak oxygen uptake (VO_2_, mL/kg/min), and ventilatory efficiency for carbon dioxide (VE/VCO_2_) at the anaerobic threshold. Breeze 7.2.0.64 SP7 and CardioControl Workstation Software last accessed on 1 September 2024 were used to process CPET data. These outcomes were risk-stratified and classified as low, intermediate, and high risk using local guidelines, with thresholds of AT < 10 mL/kg/min, Peak VO_2_ < 15 mL/kg/min, and VE/VCO_2_ at AT > 34 indicating higher risk [12,21]. As there is no ovarian-cancer-specific data, these thresholds are based on outcomes from other elective intra-abdominal surgery and POETTS-recognised thresholds [12,13].

### 2.3. Outcomes

Primary outcomes included overall survival (OS) and recurrence-free survival (RFS). Overall survival was defined as time of diagnosis to death [22]. Recurrence was defined as radiologically or histologically proven disease recurrence and was further categorised as local and distant metastasis [23]. Subgroup analyses for survival were performed for patients with advanced stage IIIB-IV disease.

### 2.4. Statistical Analyses

Means with standard deviations or medians and interquartile ranges were used to summarise continuous variables. Categorical variables were described using frequencies and percentages. Non-parametric tests, such as the Mann–Whitney U test, were used to analyse continuous data, including pairwise comparisons when relevant. Pearson’s chi-squared test and Fisher’s exact test were employed for categorical data analysis. Logistic regression models were applied to binary outcomes, adjusting for potential confounding factors. The Kaplan–Meier method was applied to estimate overall survival and recurrence-free survival according to CPET parameters, and cumulative survival between the groups was compared using the log-rank test. Cox proportional regression with Hazard’s ratio were used to estimate differences in overall survival and recurrence-free survival according to CPET parameters, after adjusting for other covariates, which included age, BMI, ASA, ECOG, FIGO staging, cytoreductive status, and CCI scores. Statistical tests were two-tailed, with significance defined at *p* < 0.05. IBM SPSS Statistics for Mac, version 29.0.2.0 (IBM Corp., Armonk, NY, USA), was used for data analysis.

## 3. Results

**Figure 1:**
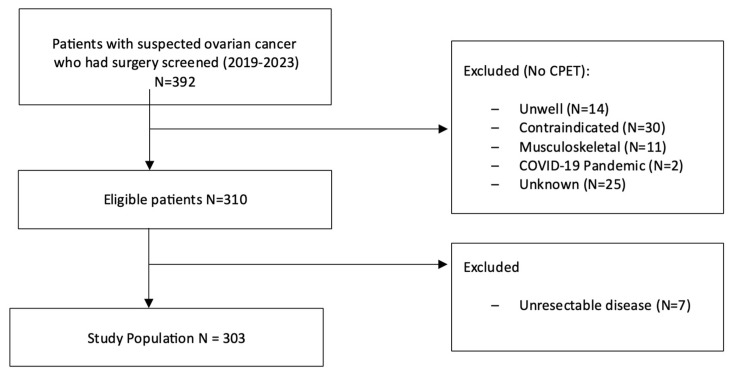
Patient selection.

A total of 392 patients underwent surgery for ovarian cancer at the Northern Gynaecological Oncology Centre between 2019 and 2023. Of these, 82 did not have CPET, and in 7 patients cytoreduction could not be performed due to unresectable disseminated disease; these patients were therefore excluded from this study (Figure 1). Our final cohort included 303 patients, with baseline and clinical characteristics presented in Table 1. The median age of the study population was 64 years, with the majority having an ECOG performance score of 0–1 (92.1%). Most patients (79%) were diagnosed with advanced-stage disease (III–IV) with high-grade serous adenocarcinoma being the most common histologic subtype (73.3%).

Fifty-five patients had a staging laparotomy for an isolated pelvic mass; of these, forty-six had a final diagnosis of stage 1 ovarian cancer. Of the remaining 248 patients, 52.8% underwent primary cytoreductive surgery, and 47.1% underwent interval cytoreductive surgery. Complete cytoreduction rates between the primary and interval debulking groups were 80.1% and 88.8%, respectively.

Of the 310 patients who attempted CPET, 95% successfully completed the test. Thirteen patients stopped the test prematurely before reaching AT mainly due to fatigue (N = 5), musculoskeletal pain (N = 4), syncope (N = 1), discomfort due to prolapse (N = 1), and unknown causes (N = 1), and AT could not be measured in one patient due to software problems. CPET outcomes are detailed in Table 2. In total, 109 patients (36.0%) had a peak VO_2_ of less than 15 mL/kg/min, 78 patients (25.7%) had a VE/VCO_2_ at AT of >34, and 86 patients (28.4%) had an anaerobic threshold less than 10 mL/kg/min. Around 34% of the patients were categorised as being at high risk for perioperative morbidity and mortality.

The associations between CPET parameters and baseline and clinical characteristics are shown in Table 3. A VO_2_ peak < 15 mL/Kg/min had a significant association with advanced age (*p* = 0.005), CCI ≥ 3 (*p* = 0.005), BMI > 30 kg/m^2^ (*p* < 0.001), ASA > 2 (*p* < 0.001), FIGO staging (*p* = 0.035), and poorer performance status (*p* = 0.009). Similarly, VE/VCO_2_ at AT > 34 was also significantly associated with advanced age (*p* < 0.001), BMI > 30 kg/m^2^ (*p* = 0.027), and higher ASA of 3 (0.004). AT < 10 mL/kg/min was significantly associated with BMI > 30 kg/m^2^ (*p* < 0.001) and an ASA of 3 or higher (*p* < 0.001).

### 3.1. CPET Parameters and Overall Survival

**Figure 2:**
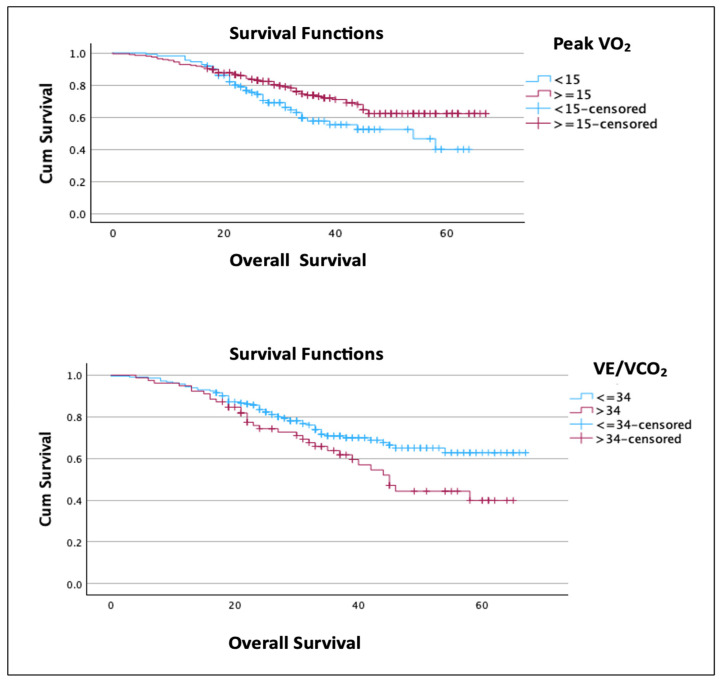
Overall survival according to CPET variables.

The mean estimated survival of the study population was 50 months, with a 1-year survival of 92%. Survival analysis showed that low-risk CPET performance parameters, including VO_2_ peak ≥ 15 and VE/VCO_2 at AT_ ≤ 34, were significantly associated with improved survival. The mean overall survival of peak VO_2_ ≥ 15 was 52 months vs. 45 months for the peak VO_2_ < 15 group (*p* = 0.021). In the low- and high-risk VE/CO_2_ groups (≤34 vs. >34), the mean survival was 52 and 44 months, respectively (*p* = 0.024) (Figure 2). After multivariate analyses, which corrected for other characteristics, including age, BMI, ECOG, ASA, CCI, FIGO staging, and cytoreductive status, peak VO_2_ remained significantly associated with overall survival (95% CI 0.35–0.95, *p* = 0.032); however, VE/VCO_2_ did not retain its statistical significance (*p* = 0.097). There was no association between CPET outcomes and 1-year survival.

### 3.2. Subgroup Analysis

**Figure 3:**
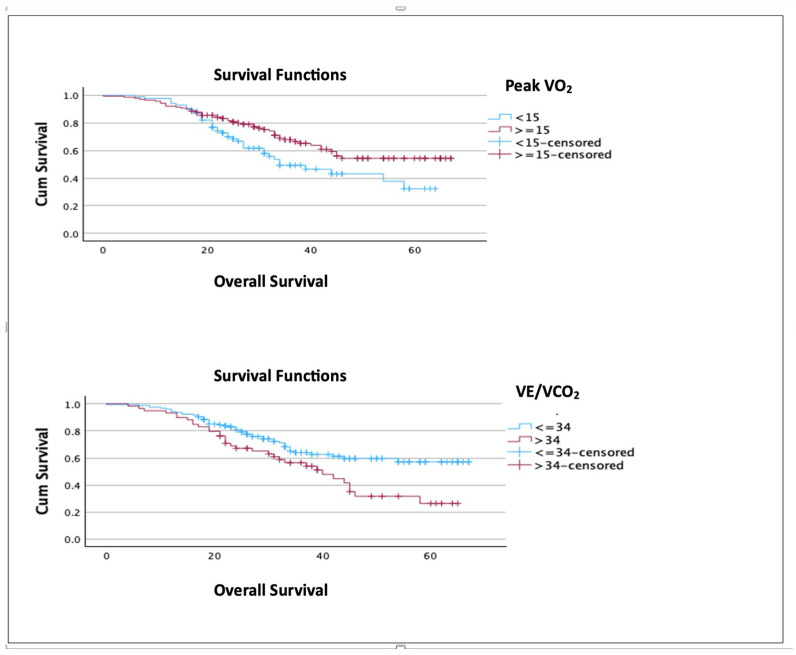
Subgroup analysis (IIIB to IVB) showing overall survival according to CPET variables.

A subgroup analysis of advanced-stage ovarian disease (IIIB to IVB) showed that both VO_2_ peak ≥ 15 (*p* = 0.017) and VE/VCO_2 at AT_ ≤ 34 (*p* = 0.009) were significantly associated with improved survival. The mean OS in peak VO_2_ groups was 49 months for the peak VO_2_ ≥ 15 group vs. 40 months for the peak VO_2_ < 15 group. In the VE/CO_2_ groups, the mean OS was 49 vs. 39 months (≤34 vs. >34) (Figure 3). Both retained their statistical significance after multivariate analysis, with *p* = 0.008 (95% CI 0.291–0.833) for VO_2_ peak and *p* = 0.025 (95% CI 1.072 to 2.829) for VE/VCO_2 at AT_.

### 3.3. CPET Parameters and Recurrence-Free Survival

VE/VCO_2 at AT_ was associated with recurrence-free survival in univariate analyses (*p* = 0.008), with a higher VE/VCO_2_ being associated with an earlier recurrence at 35 months vs. 42 months (>34 vs. ≤34). However, after multivariate analyses, which corrected for other characteristics including age, BMI, ECOG, ASA, CCI, FIGO staging, and cytoreductive status, VE/VCO_2_ did not retain its statistical significance (*p* = 0.092). VO_2_ peak (*p* = 0.119) and AT (*p* = 0.218) were not associated with recurrence-free survival. Subgroup analyses of advanced-stage disease (IIIB-IV) did not show any significant associations between CPET parameters and recurrence-free survival.

## 4. Discussion

CPET is primarily considered an anaesthetic tool for preoperative risk stratification and perioperative anaesthetic planning. However, it is increasingly being recognised as an objective measure to aid post-operative risk stratification and possibly predict long-term outcomes [13,24]. We present the first study to assess the association between objective cardiovascular fitness measured by CPET parameters and survival of ovarian cancer patients. In our study, we demonstrate that overall survival is associated with VO_2_ peak and VE/VCO_2_ at AT, but we did not show an association with AT.

In ovarian cancer, there is a complex relationship between the often-extensive disease burden and its effects on nutritional and performance status. This will influence objective assessment of the cardiorespiratory system under stress using CPET in the preoperative setting [7,8,25,26]. It is logical to assume that women with lower cardiorespiratory fitness have lesser ability to withstand surgical stress and are at an increased risk of postoperative morbidity and mortality [8]. However, relating fitness to long-term outcomes also has its appeal. Studies in other cancer areas have assessed the association between pre-operative fitness and survival outcomes, with the majority being performed in cardiothoracic surgery [27,28]. Recent systematic reviews and meta-analyses by Arbee-Kalidas et al. in 6450 patients undergoing lung cancer resections concurred with our findings, indicating that VO_2_ peak >15 mL/kg/min was associated with reduced mortality [OR: 0.55, 95% CI: 0.28–0.81]. They also demonstrated an inverse relation between VO_2_ peak as a continuous variable and mortality but did not show an association between AT nor VE/VCO_2_ and survival. Heterogenicity and non-standardised exercise regimens used for CPET were the main limitations of this study [27]. A study of 95 colorectal cancer patients assessed CPET outcomes and 1-year mortality after surgery and reported that the 7 patients who died had a VO_2_ peak of <10.6 mL/kg/min. However, the study was not adequately powered to establish a significant relation between peak VO_2_ and mortality [28].

Unfortunately, most of the other studies thus far focussed on 30- and 90-day mortality [13]. A systematic review by Moran et al. of 37 studies included patients undergoing major intra-abdominal surgery. They showed a significant association between VO_2_ peak and 90-day survival after abdominal aneurysm surgery and hepatic surgery. In addition, AT was also significantly associated with short-term mortality in hepatic, vascular, colorectal, and pancreatic surgery. Only four of the included studies in the review assessed mortality at one year, and another four studies assessed the relationship between CPET and mortality >2 years after surgery. These studies all found different correlations among CPET parameters VO_2_ and AT and survival, but assessed heterogenous populations of benign and malignant diseases, with the majority being vascular or hepatic surgery. Importantly, there was a lack of uniformity in the CPET parameter thresholds assessed [13].

In our study, we did not show an association between CPET parameters and 1-year mortality, but as there were only 16 deaths, these results need to be interpreted with caution. Furthermore, Jensen et al. assessed the association between peak VO_2_ and survival of 5131 middle-aged Danish men over a follow-up period of 44 years, where they found that VO_2_ peak was significantly associated with all-cause mortality and death from several different cancers [29]. Despite the different timing of cardiorespiratory fitness measurements, it does underline the potential of VO_2_ peak as a predictor for long-term outcomes.

VO_2_ peak is a measurement that reflects the maximum VO_2_ patients feel comfortable to achieve. The ideal measurement is VO_2_ max, which is the maximum rate of oxygen uptake and utilisation by the body that a patient can possibly achieve during intense exercise. However, achieving VO_2_ max can feel unpleasant and causes symptoms such as nausea, dizziness, and even anxiety or feelings of suffocation. Therefore, VO_2_ peak is the approximate measure most used when evaluating clinical patients, despite being a lower value than a VO_2_ max would be. However, a VO_2_ peak of ≥15 mL/kg/min seems to identify those patients who can comfortably meet the increased metabolic demands of ovarian cancer and its treatments, which may explain the improved survival outcomes found in our study [30]. Other hypotheses may be that this is linked to tumour biology, chemotherapy response, or the patient’s micro-environment. Future studies are needed to further delineate these underlying mechanisms.

VE/VCO_2_, at the anaerobic threshold, is a measure of ventilatory response to exercise at the anaerobic threshold. It is determined at submaximal exercise levels, and so it is more likely to be achieved by patients than VO_2_ max. Due to the complex interplay between the cardiorespiratory systems in meeting increased metabolic demands, whilst VE/VCO_2_ appears to predominately measure ventilator function, it will be impacted by the cardiac system as well, and raised VE/VCO_2_ has been associated with poorer outcomes in patients with chronic heart failure [31]. Thus, a raised VE/VCO_2_ at the anaerobic threshold, as with a low peak VO_2_, may indicate a patient is unable to meet the metabolic demands of long-term recovery from ovarian cancer and its treatment.

Interestingly, we also showed that VE/VCO_2_ > 34 at AT was associated with poorer overall survival in a subgroup of patients with advanced-stage ovarian cancer. Other studies assessing ventilatory inefficiency through VE/VCO_2_ have been equivocal. A large study by Wilson et al. showed that increased VE/VCO_2_, measured at AT or if AT was unidentified as the lowest AT achieved in the test, was associated with decreased survival at 90 days, 2 and 5 years after surgery for colorectal cancer surgery, irrespective of metastatic spread at time of surgery. They hypothesised that this association may be explained by underdiagnosed underlying abnormalities such as heart failure, pulmonary hypertension, COPD, or interstitial pulmonary disease, which may be similar for our population [32]. However, they used a modified VE/VCO_2_ cut-off of 39, which is higher than what is commonly used in practise. The previously mentioned review by Arbee-Kalidas et al. showed a significant association between VE/VCO_2_ and decreased post operative morbidity but not with overall survival in most included studies [27]. These findings are consistent with our findings and also in line with our previous study, which demonstrated an association between VE/VCO_2_ at AT and postoperative morbidity after primary ovarian cancer surgery but not with mortality [8].

In our study, no CPET parameter was associated with disease recurrence or recurrence-free survival. As we are the first to assess this finding, further studies are needed to confirm this. Cardiorespiratory fitness is not an established risk factor in ovarian cancer recurrence [1]. However, disease recurrence is linked to chemotherapy response score and tumour biology, which may still be reflected in a patient’s fitness [1]. The ongoing Lifestyle Intervention for oVarian cancer Enhanced Survival (LIVES –NRG/GOG0225) trial, which was designed to test the relation between physical activity and recurrence-free survival in ovarian cancer, will shed more light on this area once its results become available [26].

Despite the growing burden of obesity and physical inactivity worldwide, physical activity and BMI are still not consistently recognised as an important modifiable factor within the ovarian cancer population [1,33,34,35]. Counselling practices are insufficient and incongruent with the needs of the gynaecological cancer patient population [36,37]. In addition, prehabilitation and rehabilitation programmes are still a long way from being incorporated into standard clinical practise [1,38,39]. Prehabilitation programmes show promising results in terms of improving short-term morbidity and possibly mortality; however, sustainability and effect on long-term outcomes are yet to be assessed [38]. Equally, rehabilitation programmes for ovarian cancer patients after treatment are more aimed at improving the quality of life of survivors [40].

We believe that CPET is a unique objective tool that can be used to identify patients at risk of poorer outcomes following ovarian cancer treatment, in addition to its central role within the anaesthetic assessment. Future studies should focus on further delineating this relationship to identify patients at risk of poorer outcomes. In addition, uniform cut-off values of CPET parameters are needed as there is a large discrepancy in the current literature. However, this may pose some difficulty as there is no “one size fits all”, and values may need to be adjusted to different surgical populations [10,15,32].

This is the first study to assess the relationship between CPET parameters and overall and recurrence-free survival in ovarian cancer patients. The strengths of this study include the large patient cohort and the correction for possible confounding factors and sub-analyses. However, this study is limited by its retrospective design and heterogenous study cohort, which included both patients who had surgery in primary and interval settings after neoadjuvant chemotherapy. Another limitation of our study is the inability to account for other confounding variables such as the response to chemotherapy and genetic mutation status, which could independently influence patient outcomes and may interact with the variables included in our analysis. Chemotherapy is known to influence a patient’s fitness by causing fatigue, anaemia, loss of appetite, and oral ulcers, leading to poorer nutritional intake protein depletion and muscle wasting but may simultaneously have a positive effect on disease burden [1,41,42]. In addition, patient selection for interval cytoreductive surgery, due to disease distribution and extent, and patient fitness should not be overlooked. Through sub-analyses, we have tried to take this into account, but larger case series are needed to draw separate conclusions for these different groups.

## 5. Conclusions

We are the first to demonstrate the association between fitness as measured by the CPET parameters peak VO_2_ and VE/VCO_2_ at AT and survival outcomes. In our study, VO_2_ peak ≥ 15 was significantly associated with improved OS, and VE/VCO_2_ at AT ≤ 34 was associated with improved survival in patients with advanced-stage disease. AT may possibly have a predictive value in a subset of patients but requires further research. More studies are needed to delineate the relationship between CPET parameters and long-term outcomes and to assess to what extent these effects are modifiable to improve survival and quality-of-life outcomes.

## Figures and Tables

**Table 1:** Baseline and clinical characteristics of the study population.

Characteristics	Study Population N = 303	Percentage (%)
Age in years (median, range)	64 (30–85)	
ECOG		
0	167	(55.1%)
1	112	(37.0%)
2	20	(6.6%)
3	4	(1.3%)
BMI (kg/m^2^)		
Underweight (<18.5)	5	(1.7%)
Normal (18.5–24.9)	109	(36.0%)
Overweight (25–29.9)	110	(36.3%)
Obese (30–39.9)	66	(21.8%)
Morbidly obese (>40)	12	(4.0%)
Not recorded	1	(0.3%)
Charlson Comorbidity Index		
Low (0)	158	(52.1%)
Medium (1–2)	106	(35.0%)
High (3–4)	29	(9.6%)
Very high (>4)	10	(3.3%)
Smoking		
Yes	30	(9.9%)
No	271	(89.4%)
Unknown	2	(0.7%)
ASA Score		
1	9	(3.0%)
2	163	(53.8%)
3	125	(41.3%)
4	1	(0.3%)
Unknown	5	(1.7%)
Stage		
I	46	(15.2%)
II	18	(6.0%)
IIIa	15	(5.0%)
IIIb	15	(5.0%)
IIIc	114	(37.6%)
IVa	25	(8.3%)
IVb	70	(23.1%)
Histology		
High-grade serous	222	(73.3%)
Mucinous	15	(5.0%)
Clear cell	14	(4.6%)
Granulosa cell	3	(1.0%)
Low-grade serous	15	(5.0%)
Endometroid	12	(4.0%)
Mixed	8	(2.6%)
Others	14	(4.7%)
Surgery		
Staging laparotomy	55	(18.1%)
Primary surgery	131	(52.8%)
Interval surgery	117	(47.1%)
Primary cytoreductive surgery		
Complete	105	(80.1%)
Optimal	20	(15.3%)
Suboptimal	6	(4.6%)
Interval cytoreductive surgery		
Complete	104	(88.8%)
Optimal	8	(6.8%)
Sub-optimal	5	(4.2%)
OS in months (median, range)	32	(0–67)
Survival		
<1 year	16	(5.2%)
≥1 year	287	(94.7%)
Recurrence		
<1 year	24	(7.9%)
≥1 year	120	(39.6%)
No recurrence	159	(52.5%)

ECOG: Eastern Cooperative Oncology Group; BMI: body mass index; ASA: American Society of Anesthesiologists; OS: overall survival.

**Table 2:** CPET outcomes of the study population.

CPET Outcomes	Study Population N = 303	Percentage (%)
VO_2_ peak (mL/kg/min)		
<15	109	(36.0%)
≥15	193	(63.9%)
Unknown	1	(0.3%)
VE/VCO_2_		
≤34	212	(70.0%)
>34	78	(25.7%)
Unknown	13	(4.3%)
Anaerobic threshold (mL/min)		
<10	86	(28.4%)
≥10	204	(67.3%)
Unknown	13	(4.3%)
Risk category		
Low	121	(39.9%)
Intermediate	38	(12.5%)
High	104	(34.3%)
Unknown	40	(13.2%)

**Table 3:** Association between baseline characteristics and CPET.

	VO_2_ Peak <15 vs. ≥15 (mL/kg/min)	VO_2_ Peak Continuous (mL/kg/min)	VE/VCO_2_ ≤34 vs. >34	VE/VCO_2_ Continuous	AT <10 vs. ≥10 (mL/kg/min)	AT Continuous (mL/kg/min)
**Age**	0.005 *	Not performed	<0.001 *	Not performed	0.930	Not Performed
**ECOG** **(0–4)**	0.009 *	Not performed	0.025 *	Not performed	0.294	Not performed
**CCI** **<3 vs. ≥3**	0.005 * <3: 33.0% < 15 ≥3: 56.4% < 15	<0.001 *	0.061	0.038 *	0.298	0.112
**BMI** **<30 vs. ≥30**	<0.001 * <30: 25.0% < 15 ≥30: 68.8% < 15	<0.001 *	0.027 * <30: 29.9% > 34 ≥30: 16.6% > 34	<0.001 *	<0.001 * <30: 22.1% < 10 ≥30: 52.7% < 10	<0.001 *
**ASA** **≤2 vs. >2**	<0.001 * ≤2: 19.7% < 15 >2: 58.4% < 15	<0.001 *	0.004 * ≤2: 20.7% > 34 >2: 35.8% > 34	<0.001 *	<0.001 * ≤2: 18.3% < 10 >2: 46.1% < 10	<0.001 *
**FIGO Stage** **(I–IV)**	0.035 *	Not Performed	0.413	Not Performed	0.303	Not Performed

*: *p* < 0.05, ECOG: Eastern Cooperative Oncology Group, ASA: American Society of Anesthesiologists, CCI: Charlson Comorbidity Index, BMI: body mass index.

## Data Availability

The data presented in this study are available in this article.
